# Efficacy of acupuncture combined with heat-clearing prescription on patients with pre-diabetes caused by excessive heat with yin deficiency

**DOI:** 10.12669/pjms.41.6.12153

**Published:** 2025-06

**Authors:** Zengyang Ji, Haitao Lu, Wenbin Ye

**Affiliations:** 1 Zengyang Ji Department of Endocrine, TCM Hospital of Changxing, Huzhou, Zhejiang Province 313100, P.R. China; 2 Haitao Lu Department of Nephrology, TCM Hospital of Changxing, Huzhou, Zhejiang Province 313100, P.R. China; 3 Wenbin Ye Department of Endocrine, TCM Hospital of Changxing, Huzhou, Zhejiang Province 313100, P.R. China

**Keywords:** Acupuncture, Heat-clearing Prescription, Pre-diabetes, Excessive heat with yin deficiency

## Abstract

**Objective:**

Traditional Chinese Medicine (TCM) has been proven to have a stable therapeutic effect and low toxicity. This study explored the effect of acupuncture combined with a heat-clearing prescription on patients with prediabetes mellitus (PDM) caused by excessive heat with yin deficiency.

**Methods:**

This retrospective analysis was conducted at the Changxing County TCM Hospital. A total of 297 cases of PDM caused by excessive heat with yin deficiency was conducted using clinical records of patients treated in the hospital from February 2023 to July 2024. Patients receiving acupuncture combined with a heat-clearing prescription (TCM group, n=40) were matched based on age at a 1:1 ratio with a cohort of 40 patients receiving traditional treatment (traditional group). The primary outcome was blood glucose levels, and the secondary outcomes were pancreatic beta cell function, cytokines, quality of life (QOL), and adverse events.

**Results:**

After treatment, the decrease in blood glucose levels in the TCM group was significantly lower than the traditional group (P<0.05). The improvement of homeostasis model assessment of beta-cell function (HOMA-β), Fasting Insulin (FINS), and HOMA insulin resistance (HOMA-IR) in the TCM group was better than the traditional group (P<0.05). The decrease in inflammatory cytokine levels in the TCM group was lower than the traditional group (P<0.05). Similarly, the decrease in diabetes-specific QOL scale (DSQL) score in the TCM group was lower than in the traditional group (P<0.05).

**Conclusions:**

Compared with traditional therapies, acupuncture combined with heat-clearing prescription has a more significant effect on treating patients with PDM caused by excessive heat with yin deficiency.

## INTRODUCTION

Pre-diabetes (PDM) is defined as dysglycemia, which leads to increased blood sugar, but has not yet reached the diagnostic criteria for diabetes.^1^,^2^ Research shows that the incidence of PDM is about 15.5%, and in about 70% of cases, PDM can progress to diabetes.^2^-^4^ Studies show that PDM is reversible, and patients can delay or prevent its progression to diabetes through active exercise and a healthy diet.^3^-^5^ Therefore, timely implementation of effective intervention in PDM is crucial.^4^,^5^

Clinically, interventions such as metformin treatment, lifestyle changes, and dietary guidance are often used to control blood glucose levels in patients with PDM.^4^-^6^ However, these methods are associated with adverse effects such as recurrence and gastrointestinal side effects after discontinuation of medication.^5^,^6^ With the continuous attention and recognition of traditional Chinese Medicine (TCM), the trend of PDM treatment is shifting towards integrating TCM and Western medicine.^7^,^8^ TCM believes that the leading causes of PDM are innate endowments, overeating sweetness, emotional imbalance, and ease of movement.^7^-^9^

PDM is based on yin deficiency and dryness heat.^8^ Acupuncture and heat-clearing prescription are empirical methods formulated by our hospital according to the characteristics of PDM caused by excessive heat with yin deficiency.^9^-^12^ Acupuncture can promote metabolism, balance yin and yang, regulate qi and blood, improve the function of the immune system and neuroendocrine system, and regulate insulin sensitivity and blood glucose levels.^10^,^12^ Heat-clearing prescription can nourish yin, produce fluids, clear heat, and moisten dryness. Studies show that acupuncture and heat-clearing prescriptions have good therapeutic effects on various diseases of yin deficiency and internal heat.^9^,^11^

Zhou et al.^11^ showed that combining heat-clearing prescription and acupuncture significantly affected chronic pelvic inflammatory disease with damp-heat and blood-stasis syndrome. Ma et al.^12^ confirmed the effect of heat-clearing prescription combined with acupuncture in treating insomnia of restless type with yin deficiency and blood deficiency in the elderly. However, the efficiency of TCM in treating PDM caused by excessive heat with yin deficiency is still unclear due to the limited number of available reports.^9^,^10^

This retrospective analysis aimed to evaluate the effectiveness of acupuncture combined with heat-clearing prescription in treating patients with PDM caused by excessive heat with yin deficiency. It specifically focuses on blood glucose, pancreatic beta cell function, and cytokines levels, as well as quality of life (QOL) and adverse events.

## METHODS

This single-center retrospective analysis was conducted in the Endocrinology Department of Changxing County TCM Hospital. Clinical records of 297 patients with PDM caused by excessive heat with yin deficiency, treated from February 2023 to July 2024, were retrospectively analyzed.

### Ethical Approval:

The Medical Ethics Committee of Changxing County TCM Hospital has approved this study (Approval No.: 2024-063-01); Date: December 25^th^, 2024. Due to the study’s retrospective nature, the Medical Ethics Committee of Changxing County Hospital of TCM has waived informed consent. All data were securely stored and kept confidential throughout the entire research process.

### Inclusion criteria:

- Diagnosis of PDM caused by excessive heat with yin deficiency, made by combining TCM and Western medicine.^1^,^7^,^8^
- Age range: 18-75 years old.
- Complete clinical data.

### Exclusion criteria:

- History of diabetes (except gestational diabetes).
- Patients with vital organ dysfunction (such as heart, lung, liver, and kidney).
- Patients with immune system disorders (such as rheumatoid arthritis, and systemic lupus erythematosus).
- Breastfeeding and pregnant women.
- History of glucocorticoids, beta blockers, thiazide diuretics, and niacin use.
- Individuals with malignant tumors (such as lung cancer, and stomach cancer).

### Traditional treatment method:

### Health Education:

- Patients were provided customized intervention plans based on their lifestyle, diet, and daily habits.
- Regular online lectures were conducted to educate patients on PDM caused by excessive heat with yin deficiency (occurrence, development, treatment, and prognosis of diabetes) to give patients a comprehensive understanding of PDM caused by excessive heat with yin deficiency and the importance of the treatment.
- A patient social software communication group was established to share scientific articles about PDM caused by excessive heat with yin deficiency to promote communication and interaction between doctors and patients and answer patients’ questions about the condition.
- Two weekly phone follow-ups for patients unfamiliar with social media were conducted to guide them in maintaining a good lifestyle.

### Dietary management:

- Patients were guided to follow the principle of a low sugar and low salt diet and eat timely, small, and frequent meals.
- To enhance satiety, Patients were guided to prioritize consuming foods rich in crude fiber, such as brown rice, noodles, and vegetables.
- Patients were encouraged to quit smoking and drinking and to pay attention to controlling the total calorie intake of daily food. Each meal’s fat, carbohydrate, and protein content should be 30%, 55%, and 15%, respectively.
- Patients were encouraged to strictly control the intake of sugary drinks, maintain good eating habits, and control weight.

### Sports management:

A reasonable physical exercise plan was developed based on each patient’s condition. The plan included moderate-intensity aerobic exercises such as jogging, walking, Tai Chi, Baduanjin, and ball games. The exercise time was about 45 minutes daily, five times or more per week. Patients were encouraged to share their exercise results through social media every day to strengthen their compliance.

### Psychological education:

Patients maintained contact with doctors throughout the entire treatment period and received timely psychological support, enabling them to face the disease optimistically and cooperate with treatment seriously.

### Medications

Oral metformin (Bristol Myers Squibb, Shanghai China; Specification: 0.5g × 10 pieces) was administered 0.25 g/time, three times/day. Subsequently, the medication dosage was increased to 0.5 g/time and three times/day based on the patient’s condition and tolerance. Metformin was taken continuously for three months.

### TCM method:

The TCM group was treated with acupuncture combined with a heat-clearing prescription, performed in addition to the traditional treatment.

- *Acupuncture*: The disposable sterile acupuncture needle (0.3 mm × 50 mm) was selected. the principal acupoints include Pishu (BL20), Weishu (BL21), Ganshu (BL18), Zhangmen (LR13), Zhongwan (RN12), Qimen (LA14), and Zhiyang (DU9), and the supplementary acupoints will be Taichong (LR3), Hegu (L14), Zusanli (ST36), Yinlingquan (SP9), Sanyinjiao (SP6), Fenglong (ST40), Shangjuxu (ST37), and Xiajuxu (ST39). All acupoints will be stimulated by neutral reinforcement and reduction of movement. The needle was left for 30 minutes. Treatment was done once a day, five times a week.
- *Heat-clearing prescription*: The medicinal formula included a mixture of Schisandra Chinensis (Wu Weizi) 6g, Coptis Chinensis (Huang Lian) 6 g, Dark Plum (Wu Mei) 10 g, Poria (Fu Ling) 12 g, Dendrobium Herba Dendrobii (Shi Hu) 15 g, Ophiopogonis Radix (Mai Dong) 15g, Salvia miltiorrhiza (Dan shen) 15g, Pueraria lobata (Ge Gen) 15 g, Radix Astragali (Huang Qi) 15 g, rehmannia root (Di Huang Gen) 20 g, which were boiled in water. A total of 400 milliliters of preparation was collected, and patients were instructed to continuously take it in portions in the morning and evening for three months.

### Primary Outcome:

The primary outcome was levels of glucose (FBG), two-hour plasma glucose (2hPG), and hemoglobin A1c (HbA1c) after 12 weeks of treatment. A Roche blood glucose meter was used to detect the above indicators.

### Secondary outcome:

The secondary outcomes after 12 weeks of treatment were as follows:

- Pancreatic beta cell function, including homeostasis model assessment of beta-cell function (HOMA-β), Fasting Insulin (FINS), and HOMA insulin resistance (HOMA-IR). Radioimmunoassay was used to detect FINS, and calculate HOMA - β = 20 × FINs/(FPG-3.5) and HOMA IR = FINs × FPG/22.5.
- Cytokines, including interleukin -6 (IL-6), IL-8, and tumor necrosis factor-alpha (TNF-α) levels. The enzyme-linked immunosorbent assay (ELISA) was used to detect the levels of the above indicators, and the reagent kit was purchased from China Biyun Tian.
- QOL was assessed using the diabetes-specific QOL scale (DSQL), which has a total score of 100 points. The lower score indicates better QOL.
- Adverse events, including needle bending, needle stagnation, fainting or bleeding during needle insertion, and adverse reactions to major primary organs such as the heart, liver, kidney and brain.

### Statistical analysis:

The normality of the distribution of continuous variables was confirmed using the Shapiro-Wilk test. Mean ± standard deviation (SD) was used for normally distributed data. Paired samples were analyzed using a paired t-test, and independent samples were analyzed using a t-test. For non-normally distributed data, the intergroup comparison was performed using nonparametric methods such as the Wilcoxon rank sum test, represented by the interquartile range (IQR) deviation. The descriptive analysis used frequency or ratio (%) to report categorical variables. The Chi-square test or Fisher’s exact test was used to evaluate the differences in count variables. SPSS software (version 22.0; IBM, Armonk, NY) was used for this study. Bilateral P<0.05 has statistical significance.

## RESULTS

**Fig.1:**
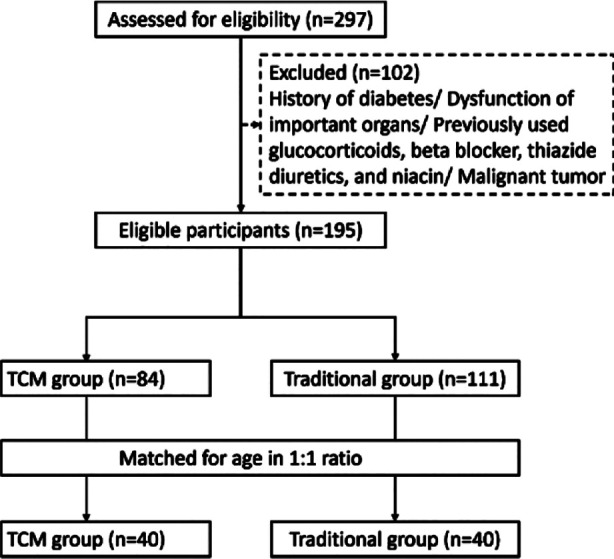
Patient screening process diagram. TCM, Traditional Chinese Medicine.

This retrospective study included 80 patients, 46 males and 34 females. The age range was 35-75, with an average age of 53.4 ± 8.3. Patients receiving acupuncture combined with a heat-clearing prescription (TCM group) were matched based on age at a 1:1 ratio with a cohort of 40 patients receiving traditional treatment (traditional group), 40 patients per group (Fig.1). No significant demographic differences were observed between the two groups (P>0.05) (Table-I).

**Table-I:** Comparison of clinical characteristics between two groups.

Characteristics	TCM group (n=40)	Traditional group (n=40)	t/χ^2^	P
Male (yes), n (%)	25 (62.5)	21 (52.5)	0.818	0.366
Age (years), mean+SD	54.7±7.7	52.1±8.7	1.390	0.168
Body Mass Index (kg/m2)	23.1±2.9	23.8±2.7	-0.998	0.321
Disease duration (months), IQR	3 (2-3.5)	3 (2-4)	-0.453	0.651
Diabetes family (yes), n (%)	9 (22.5)	6 (15.0)	0.738	0.390
Drinking history (yes), n (%)	16 (40.0)	18 (45.0)	0.205	0.651
Smoking history (yes), n (%)	20 (50.0)	17 (42.5)	0.453	0.501
Hypertension (yes), n (%)	11 (27.5)	14 (35.0)	0.524	0.469
Hyperlipidemia (yes), n (%)	20 (50.0)	24 (60.0)	0.808	0.369
Coronary heart disease (yes), n (%)	5 (12.5)	3 (7.5)	0.556	0.709^b^

b Fisher’s exact test. TCM, Traditional Chinese Medicine; BMI, body mass index; SD, standard deviation; IQR, interquartile range.

Before treatment, there was no significant intergroup difference in the baseline FPG, 2hPG, and HbA1c levels (all P>0.05). After 12 weeks of treatment, FPG, 2hPG, and HbA1c in both groups decreased significantly compared to before treatment and were significantly lower in the TCM group compared to the traditional group (all P<0.05) (Table-II).

**Table-II:** Comparison of blood glucose levels between two groups.

Item	TCM group (n=40)	Traditional group (n=40)	Z/t	P
***Before treatment***				
FPG (mmol/L)	6.54(6.36-6.77)	6.61 (6.46- 6.85)	-1.314	0.189
2hPG (mmol/L)	10.33 (9.41-10.92)	9.88 (9.40-10.59)	-0.789	0.430
HbA1c (%)	6.12±0.23	6.05±0.21	1.422	0.159
***After treatment***				
FPG (mmol/L)	4.87±0.69	5.33±0.62	-3.190	0.002
2hPG (mmol/L)	7.41 (6.71-7.61)	7.65 (7.43-8.12)	-5.270	<0.001
HbA1c (%)	5.26±0.41	5.70±0.42	-4.662	<0.001

TCM, Traditional Chinese Medicine; FPG, fasting plasma glucose; 2hPG, two-hour plasma glucose; HbA1c, hemoglobin A1c.

Before treatment, the two groups had no significant difference in baseline HOMA - β, HOMA-IR, and FINS levels (P>0.05). After 12 weeks of treatment, HOMA - β and FINS in both groups increased compared to before treatment and were significantly higher in the TCM group than in the traditional group, while post-treatment HOMA-IR levels decreased in both groups and were considerably lower in the TCM group than in the traditional group (P<0.05) (Table-III).

**Table-III:** Comparison of pancreatic beta cell function between two groups.

Item	TCM group (n=40)	Traditional group (n=40)	Z/t	P
Before treatment				
HOMA-β	76.3±13.4	74.9±15.8	0.442	0.660
HOMA-IR	3.72 (2.92-4.02)	3.68 (3.06-4.13)	-0.491	0.623
FINS (mU/L)	27.6±3.4	28.8±4.1	-1.402	0.165
After treatment				
HOMA-β	104.3±20.3	90.4±17.5	3.278	0.002
HOMA-IR	2.24±0.38	2.81±0.46	-6.132	<0.001
FINS (mU/L)	16.1±1.82	20.3±2.8	-7.955	<0.001

TCM, Traditional Chinese Medicine; HOMA-β, homeostasis model assessment of beta-cell function; FINS, fasting Insulin; HOMA-IR, HOMA insulin resistance.

**Fig.2:**
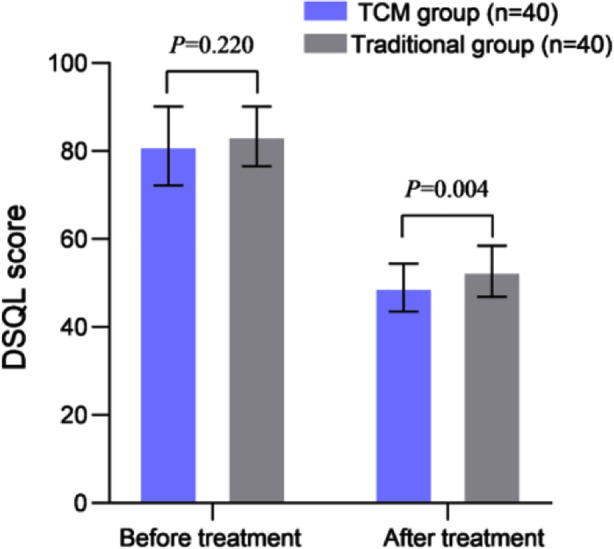
Comparison of DSQL scores between two groups. TCM, Traditional Chinese Medicine; DSQL, the decrease in diabetes-specific quality of life scale.

Before treatment, baseline IL-6, IL-8, and TNF - α levels were comparable in all groups (P>0.05). After 12 weeks of treatment, the levels of these indexes decreased in both groups and were markedly lower in the TCM group compared to the traditional group (all P<0.05) (Table-IV). The two groups had no significant difference in baseline pre-treatment DSQL scores (P>0.05). After 12 weeks of treatment, the DSQL score of both groups decreased and was considerably lower in the TCM group (P<0.05) (Fig.2). Patients in this study reported no treatment-related adverse events.

**Table-IV:** Comparison of cytokine levels between two groups.

Item	TCM group (n=40)	Traditional group (n=40)	t	P
Before treatment				
IL-6, ng/L	86.2±10.6	83.3±13.9	1.039	0.302
IL-8, ng/L	83.1±11.2	81.4±13.3	0.632	0.530
TNF-α, ng/L	87.7±10.5	84.7±13.9	0.898	0.372
After treatment				
IL-6, ng/L	21.7±4.4	29.5±5.6	-6.943	<0.001
IL-8, ng/L	29.6±5.1	37.5±6.7	-5.956	<0.001
TNF-α, ng/L	22.5±4.0	30.3±3.9	-8.713	<0.001

TCM, Traditional Chinese Medicine; IL-6, interleukin 6; IL-8, interleukin 8; TNF-α, tumor necrosis factor-alpha.

## DISCUSSION

This study explored the therapeutic effect of acupuncture combined with heat-clearing prescription on PDM caused by excessive heat with yin deficiency in patients who receive traditional treatment. The results showed that acupuncture combined with heat-clearing prescription could significantly reduce patients’ blood sugar levels, improve the function of islet β cells, reduce inflammation, and improve patients’ QOL.

This study further confirms previous research demonstrating the benefits of TCM treatment and acupuncture intervention in reducing blood sugar.^13^,^14^ The results showed that after treatment, the FPG, 2hPG, and HbA1c levels in the TCM group were lower than those in the traditional group, indicating that the combination of acupuncture and medication can effectively regulate blood glucose levels of patients with PDM caused by excessive heat with yin deficiency and may improve the effectiveness of the treatment. From the perspective of TCM meridian theory, acupuncture at acupoints such as Pishu (BL20), Weishu (BL21), Ganshu (BL18), Zusanli (ST36) may directly or indirectly regulate meridians related to the spleen, stomach, liver, and kidneys, and ensure the normal metabolism of ingested carbohydrates.^9^-^12^,^15^

A study by Li et al.^15^ confirmed that acupuncture combined with lifestyle intervention is more effective than lifestyle intervention alone in reducing the blood sugar level in patients with PDM caused by excessive heat with yin deficiency. The medicinal ingredients in the heat-clearing prescription exert multiple effects on blood sugar levels. Coptis Chinensis (Huang Lian) has the effects of clearing heat, drying dampness, purging fire, and detoxifying. The berberine component in Coptis Chinensis (Huang Lian) can improve insulin resistance, promote glucose uptake and utilization, and thus lower blood sugar.^16^,^17^

The organic acids and polysaccharides in Dark Plum (Wu Mei) have antioxidant activity, protect pancreatic beta cells, and lower blood sugar levels.^18^ Schisandra Chinensis (Wu Weizi) has the effects of astringency, tonifying qi, and generating fluids. It can regulate the body’s endocrine system and enhance the ability to regulate blood sugar.^19^ Radix Astragali (Huang Qi) is the leading herbal medicine in many patent medications for treating diabetes.^20^ A natural product called dehydroaspergilloic acid isolated from Poria (Fu Ling) cocos has been proven to be an insulin sensitizer that can induce fat conversion in vitro and reduce hyperglycemia in the mouse model of non-insulin-dependent diabetes.^21^

The flavonoids in Salvia miltiorrhiza (Dan shen) can inhibit the activity of glycogen synthase and reduce the production of glucose by the liver. It can also promote insulin secretion and utilization, thereby reducing blood sugar levels.^22^ Dendrobium Herba Dendrobii (Shi Hu), Ophiopogonis Radix (Mai Dong), Pueraria lobata (Ge Gen), and rehmannia root (Di Huang Gen), all contain various active ingredients, such as flavonoids, and polysaccharides. that were shown participate in lowering blood sugar, antioxidation, and immune regulation.^23^,^24^ Li et al.^14^ found that a heat-clearing prescription significantly improved blood sugar levels, which is consistent with the results of this study.

This study further confirmed that the combination of acupuncture and heat-clearing prescription can impact numerous aspects of glucose metabolism to achieve better control of blood glucose indicators. The results also showed that in PDM patients, the combined treatment was associated with improved function of pancreatic islet β cells, as indicated by the changes in the pancreatic beta cell function index. A study by Zhang et al.^25^ pointed out that acupuncture at certain acupoints can stimulate the nervous system, regulate the balance between sympathetic and parasympathetic nerves, and indirectly affect the secretion function of pancreatic beta cells. Similarly, herbs such as Radix Astragali (Huang Qi) and Pueraria lobata (Ge Gen) in heat-clearing prescription were shown to regulate the nervous system and protect pancreatic beta cells.^23^ Other components of the heat-clearing prescription, such as Salvia miltiorrhiza (Dan Shen), can improve microcirculation, provide sufficient nutrients and oxygen for pancreatic beta cells, and facilitate their functional repair.^23^

As demonstrated by the study by Zhang et al.,^26^ inflammation plays a vital role in the pathogenesis and progression of diabetes. In agreement with this observation, this study demonstrated that acupuncture-assisted conventional treatment can alleviate the inflammatory response in PDM patients. Previous studies showed that acupuncture can reduce inflammatory factors in the spinal cord of diabetic rats with peripheral neuropathy,^26^ Poria (Fu Ling) and Dendrobium Herba Dendrobii (Shi Hu) officinale, components of the heat-clearing prescription, have anti-inflammatory and immune-regulating effects.^21^-^24^ The current study also reported no treatment-related adverse events, which suggests that acupuncture combined with the heat-clearing prescription is safe.

The results of this study show that the DSQL score of PDM patients treated with acupuncture combined with heat-clearing prescription is significantly higher than that of the traditional group. It is plausible that this effect is due to better blood sugar control, improved pancreatic beta cell function, and reduced inflammatory reactions, which help alleviate symptoms, improve physical comfort and psychological state, and ultimately enhance the QOL of patients who receive the combined treatment.

### Clinical implications:

It embodies the characteristic TCM method of syndrome differentiation and treatment and the holistic concept. It emphasizes the advantages of using a combination of acupuncture and medicine for specific syndrome types. This approach provides new evidence for the application of TCM in the prevention and treatment of chronic diseases, further promoting the development of TCM in modern medicine.^21^,^24^,^25^,^27^,^28^

### Limitations

This is a retrospective analysis with a small sample size. A heat-clearing prescription contains many complex components, which leads to a specific herb having many pharmacological therapeutic targets. Therefore, it is difficult to fully reveal the mechanism of action of different herbs through traditional laboratory methods. TCM is characterized by syndrome differentiation and treatment, but it also has the disadvantage of poor reproducibility of therapeutic effects.

The heat-clearing prescription is fixed, which is inconsistent with the TCM concept of individualized treatment based on syndrome differentiation. In subsequent research, treatment prescriptions can be adjusted based on minor changes in organ function to ensure that each prescription is the most suitable choice for the patient’s specific situation. The small sample size and short treatment duration of 12 weeks may limit the generalizability of the results of this study. Finally, while acupoint selection is simple and effective in treating PDM caused by excessive heat with yin deficiency, applying non-standard acupoints may limit the application of this acupuncture therapy to a certain extent.

## CONCLUSION

When given in addition to the traditional treatment, acupuncture combined with heat-clearing prescription can significantly reduce the blood sugar of patients with PDM caused by excessive heat with yin deficiency, improve the function of pancreatic islet β cells, regulate the expression of cytokines, reduce the degree of inflammatory reaction in the body, and improve the QOL of PDM patients.

### Authors’ contributions:

**ZJ:** Study design, literature search and manuscript writing.

**ZJ, HL and WY:** Data collection, data analysis and interpretation. Critical Review.

All authors have read and approved the final manuscript. They are also responsible for the integrity of the study.
